# Rates of Decline in Alzheimer Disease Decrease with Age

**DOI:** 10.1371/journal.pone.0042325

**Published:** 2012-08-02

**Authors:** Dominic Holland, Rahul S. Desikan, Anders M. Dale, Linda K. McEvoy, for the Alzheimer’s Disease Neuroimaging Initiative

**Affiliations:** 1 Department of Neurosciences, University of California San Diego, La Jolla, California, United States of America; 2 Department of Radiology, University of California San Diego, La Jolla, California, United States of America; Institution of Automation, CAS, China

## Abstract

Age is the strongest risk factor for sporadic Alzheimer disease (AD), yet the effects of age on rates of clinical decline and brain atrophy in AD have been largely unexplored. Here, we examined longitudinal rates of change as a function of baseline age for measures of clinical decline and structural MRI-based regional brain atrophy, in cohorts of AD, mild cognitive impairment (MCI), and cognitively healthy (HC) individuals aged 65 to 90 years (total n = 723). The effect of age was modeled using mixed effects linear regression. There was pronounced reduction in rates of clinical decline and atrophy with age for AD and MCI individuals, whereas HCs showed increased rates of clinical decline and atrophy with age. This resulted in convergence in rates of change for HCs and patients with advancing age for several measures. Baseline cerebrospinal fluid densities of AD-relevant proteins, Aβ_1–42_, tau, and phospho-tau_181p_ (ptau), showed a similar pattern of convergence with advanced age across cohorts, particularly for ptau. In contrast, baseline clinical measures did not differ by age, indicating uniformity of clinical severity at baseline. These results imply that the phenotypic expression of AD is relatively mild in individuals older than approximately 85 years, and this may affect the ability to distinguish AD from normal aging in the very old. Our findings show that inclusion of older individuals in clinical trials will substantially reduce the power to detect disease-modifying therapeutic effects, leading to dramatic increases in required clinical trial sample sizes with age of study sample.

## Introduction

Age is the strongest risk factor for sporadic Alzheimer disease (AD) [1], [2], the most common cause of dementia. Due to growth in the proportion of elderly individuals in many societies, the prevalence of AD is increasing at an alarming rate [3]–[5]. Individuals over 85 years of age comprise the fastest growing segment of US society [6], yet it is not clear whether AD progresses in the same manner among the very old as among individuals who are affected at a younger age.

Pathologically, AD is characterized by the misfolding [7]–[9] and accumulation of amyloid-beta (Aβ) and tau proteins in insoluble aggregates, leading to the formation of amyloid plaques and neurofibrillary tangles (NFTs) [10], [11]. The increase in burden of these hallmark AD lesions in normal aging [12]–[14], combined with the increasing inability of aging cells to maintain disease proteins in benign states [15] and the exponential growth in the incidence of AD with increasing age (at least through approximately 85 years [16]), could suggest that the rate of decline in AD also increases with age. Emerging literature indicates, however, that the burden of AD lesions in individuals with AD decreases with age [17], while cognitive and structural changes might be more salient in younger rather than older individuals with AD [18]. This may reduce the ability to detect AD among the oldest old, potentially contributing to the controversy over whether AD incidence rates go up, down, or remain stable after age 85 [16].

Since the elderly population is growing, and proportionally growing faster with increasing age, it is important to better understand whether and how age interacts with the disease process to affect rates of decline. Here, we investigated whether rates of clinical decline and structural MRI-based measures of regional brain atrophy differ according to age among healthy controls (HCs) and individuals with mild cognitive impairment (MCI) or AD. We also assessed age-dependence of baseline clinical measures, and morphometric and cerebrospinal fluid (CSF) biomarkers of AD pathology. We explored the implication of the observed age-related differences in atrophic and clinical rates of decline for clinical trial design, and for disease biomarker trajectories.

## Methods

We examined participants from the Alzheimer’s Disease Neuroimaging Initiative (ADNI, www.adni-info.org). Relevant details of ADNI, including participant enrollment criteria, MR image acquisition, and CSF collection and analysis methods are provided in File S1.

### Participants

Participants enrolled in ADNI are not depressed, have a modified Hachinski score of 4 or less, and have a study partner able to provide an independent evaluation of functioning. HC participants have a Clinical Dementia Rating (CDR) of 0 [19]. Participants with MCI have a subjective memory complaint, objective memory loss measured by education-adjusted scores on Wechsler Memory Scale Logical Memory II, a CDR of 0.5, preserved activities of daily living, and absence of dementia. Participants with AD have a CDR of 0.5 or 1.0 and meet National Institute of Neurological Disorders and Stroke and Alzheimer’s Disease and Related Disorders Association criteria for probable AD [20]. Further details on inclusion/exclusion criteria are described in the ADNI 1 protocol (available at www.adni-info.org/Scientists/ProceduresManuals.aspx).

We evaluated 723 older participants (222 HC, 345 MCI, and 156 AD, Table 1), who were tested at 6- or 12-month intervals for up to 24 (AD) or 36 (HC and MCI) months. The research protocol was approved by each local institutional review board, and written informed consent was obtained from each participant. To exclude patients with early-onset AD [21], and to have a balanced age range of HC and MCI individuals with respect to the AD cohort, we restricted analysis to participants aged 65 years or older at baseline.

**Table 1:** Number of participants in each age group, for each class of measure analyzed, separately for longitudinal and baseline analyses.

Baseline Age (years)	Longitudinal						Baseline					
	MRI			Clin			CSF			Clin		
	HC	MCI	AD	HC	MCI	AD	HC	MCI	AD	HC	MCI	AD
[65 70)	5	37	20	5	45	21	4	25	10	5	47	22
[70 75)	78	77	26	86	91	42	47	52	22	97	98	43
[75 80)	59	73	34	65	87	44	38	47	27	75	89	49
[80 85)	25	65	25	28	76	37	15	34	21	31	81	42
[85 90)	12	21	–	13	30	–	5	15	–	14	30	–
Total	179	273	105	197	329	144	109	173	80	222	345	156

Clin  =  clinical measure: ADAS-Cog, CDR-SB, MMSE. CSF data were obtained at baseline for approximately half the study sample. Note that in each age bracket, age extends up to, but does not include, the highest age indicated (age groups do not overlap).

### MRI Image Processing

We preprocessed all MRI scans using image correction procedures for site-specific distortion effects updated for recent scanner changes [22]. We *qu*antified *a*natomical *r*egional *c*hange in serial MRI using Quarc [23], [24], a recently developed method from our laboratory. The longitudinal outcome measure of change with respect to baseline was calculated by directly registering each followup scan to the baseline scan. To evaluate baseline region of interest (ROI) measurements, we used a structural MRI post-processing technique that automatically delineates subcortical [25] and cortical [26] ROIs. We analyzed data from all available time points that passed local quality control (total = 2204). The number of followup scans was reduced by approximately 15% primarily due to motion artifacts, change in scanner model, or change in RF coil, as described in [22].

Methodological bias in image registration leading to elevated effect sizes remains a concern in the AD structural neuroimaging literature [24], particularly given recent reports [27]–[32] of results that are ostensibly corrected for bias but in fact, as shown in [24], remain significantly biased. Several research groups have developed robust approaches to reducing or eliminating bias [33], [34]. Our explicitly inverse-consistent approach [23] essentially eliminates potential bias by combining forward and reverse image registrations, and has been assessed vis-à-vis other approaches in [24].

### Clinical and CSF Measures

We analyzed age-dependence of baseline values and longitudinal rates of change for clinical measures used to assess disease severity–the Clinical Dementia Rating Scale, sum of boxes score (CDR-SB) [19], [35]; the cognitive subscale of the Alzheimer’s Disease Assessment Scale (ADAS-Cog); and the Mini Mental State Exam (MMSE). There were 493 AD, 1474 MCI, and 795 HC participant-visits (total = 2762).

We also examined whether values of three previously validated CSF biomarkers of AD pathology, Aβ_1–42_ (Aβ), tau, and phospho-tau_181p_ (ptau), differed with age at baseline. CSF data were available on approximately half of the participants, as indicated in Table 1.

### Mixed Effects Modeling

Longitudinal outcomes (Y_ij_ below) in all cases were change measured with respect to baseline (expressed as a percentage of baseline size for cortical thickness change and ROI volume change). MRI change-measures for each participant were calculated from directly registering each followup image to the participant’s baseline image [23].

Using all available time-points per participant, we investigated the relationship between atrophy rate and age, and rate of clinical decline and age using a linear mixed effects model [36] where, for a specific diagnostic group, the longitudinal outcome measurement Y_ij_ at time t_ij_ for participant i at followup timepoint j is Y_ij_ =  m_i_t_ij_ + b_i_a_i_t_ij_ + ε_ij_. Here, ε_ij_ is the within-participant error, assumed to be independent and identically normally distributed with zero mean and variance σ_e_^2^; m_i_ = m + γ_i_, where m is the fixed effect slope (mean rate of change for the group at the average age of the group) when controlling for age effects and γ_i_ is the corresponding between-participant random effect slope with variance σ_m_^2^; a_i_ is the mean-centered baseline age (years) of participant i, i.e., the participant’s baseline age minus the average baseline age for the group, and b_i_ = b + δ_i_, where b is the additional, age-coupled, fixed effect contribution to the slope and δ_i_ is the corresponding between-participant random effect slope with variance σ_b_^2^. We estimated the model parameters using the Matlab (R2009b) function nlmefit.

We tested the resulting linear fit for each cohort’s full data set against point estimates of annual rate of change, with 95% confidence intervals (CIs), for successive 5-year intervals in baseline age, calculated without an age-dependent term (setting the regression coefficients b_i_≡0), to assess the correctness of the estimated age-dependence of group atrophy rates. This means that, in addition to the mixed effects model just described applied to the full data set, we also assessed successive 5-year age ranges using a simpler mixed effects model, i.e., one not controlling for age, on the assumption that if there are any age-effects for 5-year windows they will be small and can be ignored. In other words, for each 5-year interval, we directly calculate the atrophy rate (as previously done in [24]) ignoring age as a covariate. All our regression models are linear in the regression parameters and all covariates, including time.

To determine the significance of the differences, between groups, of the slopes estimated for the linear dependence of atrophy rate on age, as shown in Tables 2 and 3, we used two-sample t-tests for independent samples with unequal variances (Satterthwaite’s method [37]).

We used generalized linear model regression to estimate group baseline values as a function of age for CSF, cognitive, and structural measures. We tested the resulting linear fit against point estimates from successive 5-year intervals in baseline age and plotted the results with 95% CIs.

### Sample Size Estimates

The disease effect, or maximum potentially treatable effect, for a patient cohort is the rate of decline calculated from the linear mixed effects model for the patient cohort that is in excess of that calculated for the HCs [24], and is potentially age-dependent. To estimate the sample size required in a two-arm (equal allocation) trial of a hypothetical disease-modifying treatment versus placebo, we assumed the treatment effect size of interest was 25% of the maximum potential treatment effect. We further assumed that the trial was of 24-months duration with a 6-months assessment interval, and that it would have 80% power to detect the treatment effect using a 2-sided significance level of 0.05 [24]. The power calculations, modeling linear change over time for each participant as described above, for a given age, and based on the estimated rate of decline for the patient cohorts relative to the rate of decline experienced at that age by HCs, incorporated the variance parameters for the patient cohort estimated from the mixed effects model [36]. Confidence intervals of 95% for sample size were based on 95% confidence intervals for the treatment effect size of interest.

## Results

### Annual Rates of Atrophy and Clinical Decline

**Figure 1:**
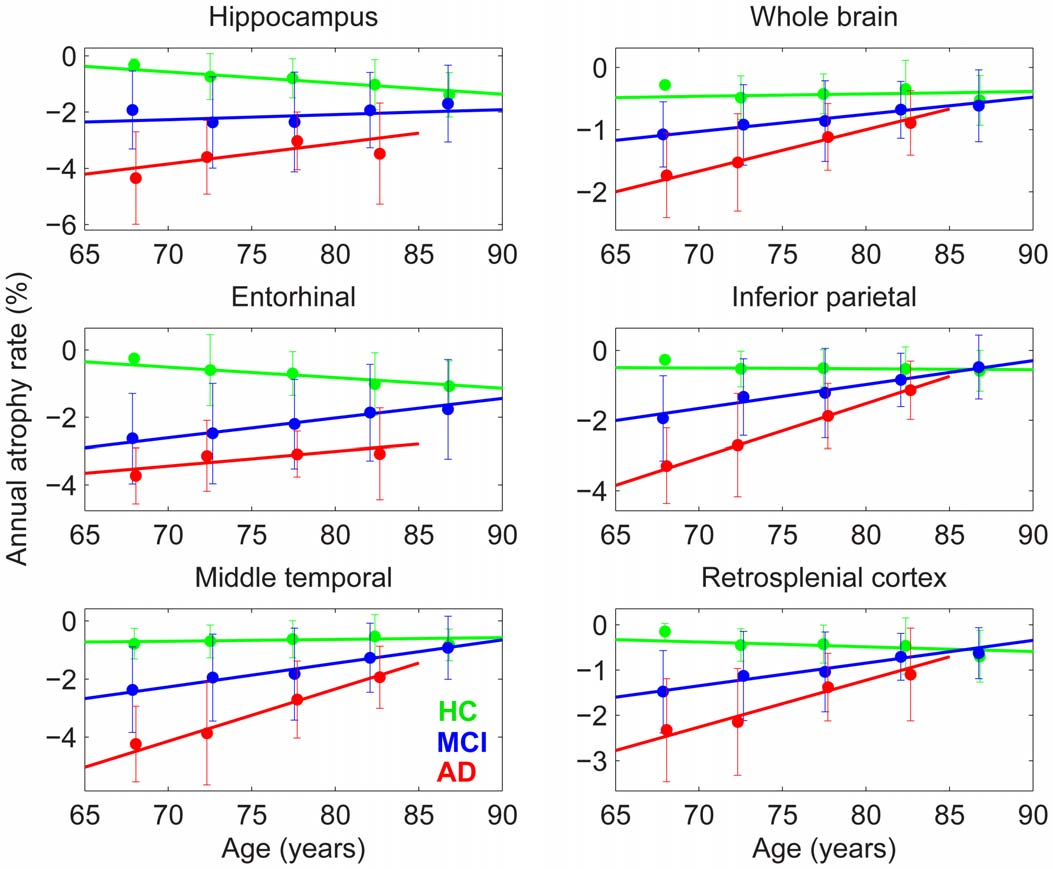
Mixed effects model fit for annual atrophy rates, allowing for linear change with age. Data points plotted, with 95% confidence intervals, are independent estimates of the atrophy rates for successive 5-year intervals for a mixed effects model fit without an age-dependent term. Slopes and p-values of the linear fits of atrophy rates with age for each cohort are shown in Table 2. Note that atrophy rate is shown by a signed value; thus a reduction in atrophy rate with age is evidenced by a positive slope in the linear fit. Legend: red  = AD; blue  =  MCI; green  = HC.

Figure 1 shows annual atrophy rates versus age for several neuroanatomical ROIs, for HC, MCI, and AD participants. The lines represent group fixed effects linear fits from mixed effects modeling of atrophy rates, including a linear dependence of atrophy rate on baseline age. The point estimates (with 95% CIs) for successive 5-year intervals are derived from independent linear mixed effect modeling without a baseline age term, and thus show the mean change experienced by participants in each 5-year age group, in each diagnostic category. The closeness of fit of the point estimates to the fixed-effects lines throughout the age range of 65 to 90 years demonstrates a strong linear dependence of atrophy rate on baseline age. Of particular significance is the pronounced decrease in atrophy rate with increasing baseline age for patient cohorts, while for HCs rates of decline either remain constant or increase with age (slopes and p-values are in Table 2). The differences in slopes between HC and patient groups were highly significant for all ROIs (p-values are in Table 2). This leads to a pattern of convergence in atrophy rates across the patient and control groups as age increases. Although reduction in atrophy rate for patient cohorts is observable in all ROIs, the degree of convergence across diagnostic groups at later ages is not uniform among ROIs. In particular, ROIs affected early in AD–the hippocampus and entorhinal cortex–maintain a significant difference in atrophy rates between HCs and AD individuals, even at 85 years, while atrophy rates for whole brain and inferior parietal cortex, for example, show almost complete convergence among diagnostic groups at this age.

**Table 2:** Slopes and p-values for annual atrophy rates as a function of age shown in Figure 1.

Measure	Slopes (% atrophy/year^2^)			P-values					
	HC	MCI	AD	HC	MCI	AD	HC-MCI	HC-AD	MCI-AD
Hippocampus	**−0.040**	0.018	**0.073**	**0.004**	0.32	**0.024**	**0.011**	**0.001**	0.13
Whole brain	0.004	**0.028**	**0.067**	0.54	**4×10^−5^**	**<10^−6^**	**0.010**	**9×10^−6^**	**0.006**
Entorhinal	**−**0.031	**0.059**	0.043	0.060	**3×10^−4^**	0.062	**0.0001**	**0.009**	0.59
Inferior parietal	**−**0.002	**0.068**	**0.155**	0.81	**<10^−6^**	**<10^−6^**	**8×10^−6^**	**<10^−6^**	**0.0005**
Middle temporal	0.006	**0.081**	**0.179**	0.59	**<10^−6^**	**<10^−6^**	**0.0001**	**<10^−6^**	**0.001**
Retrosplenial cortex	**−0.010**	**0.050**	**0.103**	0.19	**<10^−6^**	**1×10^−6^**	**1×10^−6^**	**1×10^−6^**	**0.021**

Bold underlined entries highlight slope values that significantly differ from zero. The three right hand columns give p-values for differences in slopes between cohorts. Two-sample t-test for independent samples with unequal variances (Satterthwaite’s method [37]) was used to calculate p-values for pair-wise comparisons (last three columns).

**Figure 2:**
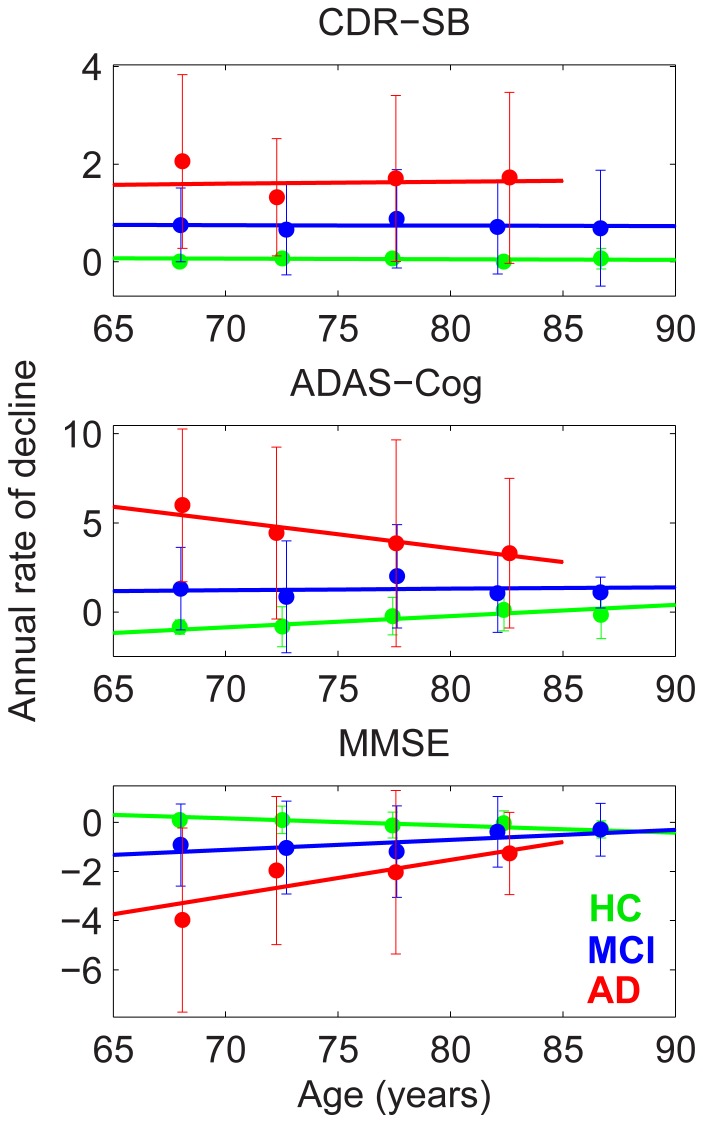
Mixed effects model fit for annual rates of clinical decline, allowing for linear change with age. Data points plotted, with 95% confidence intervals, are independent estimates of rates of change for successive 5-year intervals for a mixed effects model fit without an age-dependent term. Slopes and p-values of the linear fits for each diagnostic group are shown in Table 3. Legend: red  = AD; blue  =  MCI; green  = HC.

Figure 2 shows annual rates of decline versus age for CDR-SB, ADAS-Cog, and MMSE. Rate of decline for CDR-SB did not change with age. However, AD patients showed a decrease in rate of decline for ADAS-Cog and MMSE, whereas HCs showed a small increase in rate of decline for these measures with age, resulting in a pattern of convergence in rates of decline with advancing age (slopes and p-values are in Table 3). The differences in slopes for MMSE between HC and both patient groups, and for ADAS-Cog between HC and AD were highly significant (p-values are in Table 3).

**Table 3:** Slopes and p-values for annual rates of clinical decline as a function of age shown in Figure 2.

Measure	Slopes (decline/year^2^)			P-values					
	HC	MCI	AD	HC	MCI	AD	HC-MCI	HC-AD	MCI-AD
CDR-SB	−0.001	−0.001	0.004	0.671	0.917	0.89	0.99	0.86	0.87
ADAS-COG	**0.063**	0.009	−0.155	**0.004**	0.771	0.068	0.14	**0.013**	0.069
MMSE	**−0.029**	**0.041**	**0.147**	**0.013**	**0.025**	**0.004**	**0.001**	**0.0007**	**0.047**

Bold underlined entries highlight slope values that significantly differ from zero. The three right hand columns give p-values for differences in slopes between cohorts. P-values for the last three columns were calculated as in Table 2.

### Baseline Clinical and CSF Measures

**Figure 3:**
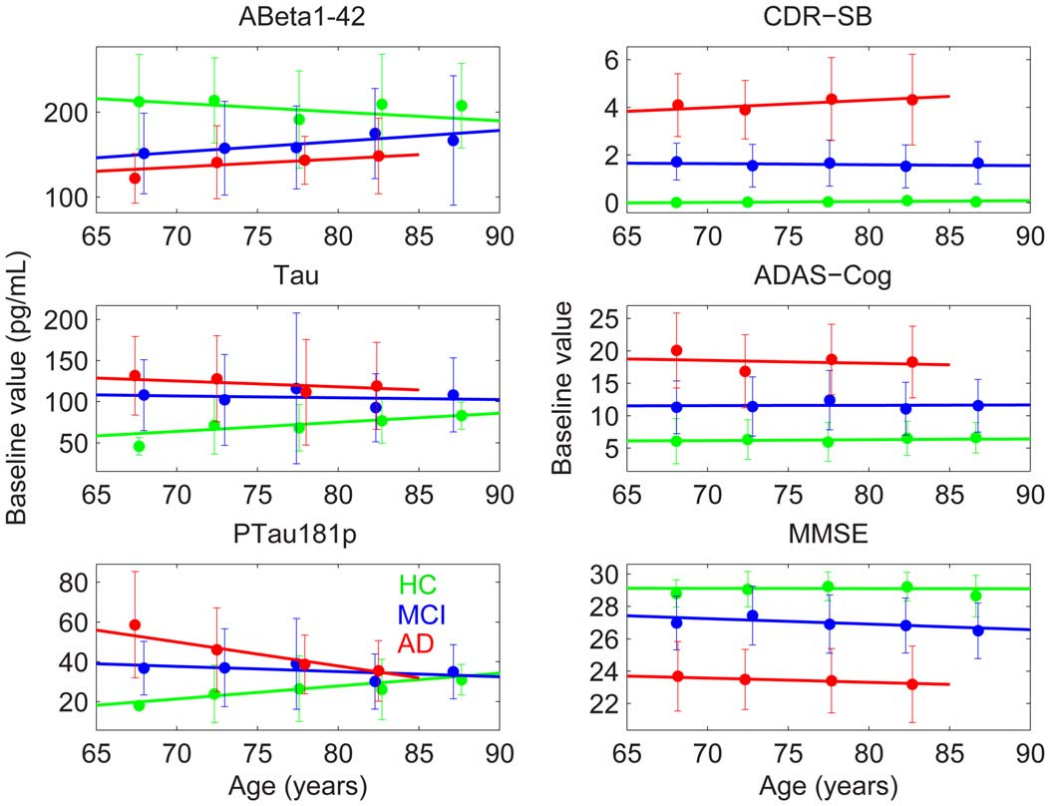
Linear fit to baseline CSF and cognitive measures, along with independent estimates of baseline measures (with 95% confidence intervals) at successive 5-year intervals. Legend: red  =  AD; blue  =  MCI; green  =  HC.

Figure 3, right column, shows the estimated relation of baseline clinical measures to baseline age, modeled with linear fits, along with the independent point estimates, with 95% CI, at successive 5-year intervals for HC, MCI, and AD participants. For all three measures, diagnostic groups are well separated across the age range, with no evidence of age-dependency on baseline clinical score.

In contrast, Figure 3, left column, indicates an age-dependency for baseline values of CSF Aβ, tau, and especially ptau. At the younger age range, AD patients show higher ptau levels than HCs. However, ptau significantly increases with age in HCs (slope  = 0.64 pg/(mL year); p = 0.035) but significantly decreases with age in AD (slope  = −1.2 pg/(mL year); p = 0.0038).

**Figure 4:**
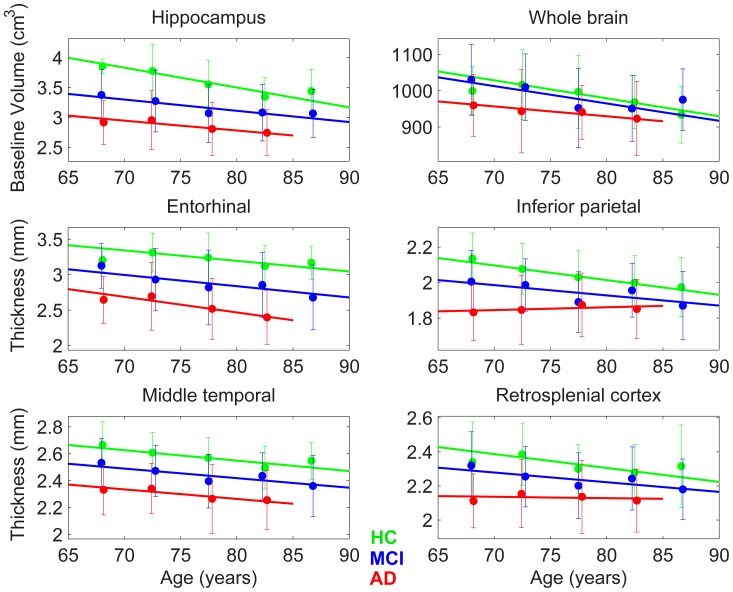
Linear fit to baseline structural MRI measures, along with independent estimates of baseline measures (with 95% confidence intervals) at successive 5-year intervals.

Plots of the age-dependence of baseline structural measures are shown in Figure 4. As expected, structures continue to atrophy as age advances (negative slopes for the HCs), and as disease advances (in general, smaller structures for more advanced disease stages at any given age). Differences in baseline structural measures between AD and HC groups decrease with advancing age for whole brain, inferior parietal lobule, and retrosplenial cortex.

### Rate of Progression to AD

**Figure 5:**
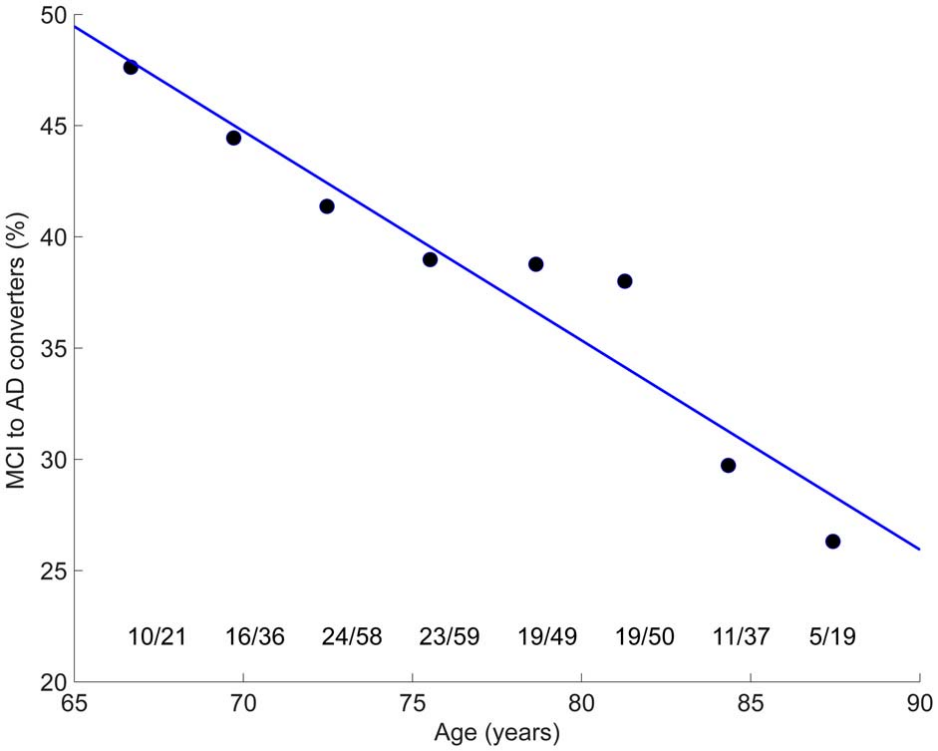
Data points show percentage, for successive 3-year age groups, of participants who progressed to dementia within 36-months from baseline. Linear fit to data has slope  = −0.94 [%/year] (standard error  = 0.11, p = 0.00016). Ratios give the number of those who developed dementia to the total number of MCI participants, for each 3-year age window.

Of 329 MCI participants with longitudinal clinical data aged 65 years and older, 127 participants were known to progress to AD within 36-months from baseline. Data points in Figure 5 show the percentage of these converters for successive 3-year baseline age intervals. Linear fit to the data has slope = −0.94%/year (standard error = 0.11, p = 0.00016). Between the ages of 65 and 90 years, the estimated progression rate approximately halves: from almost 50% to 25%.

### Sample Size Estimates

**Figure 6:**
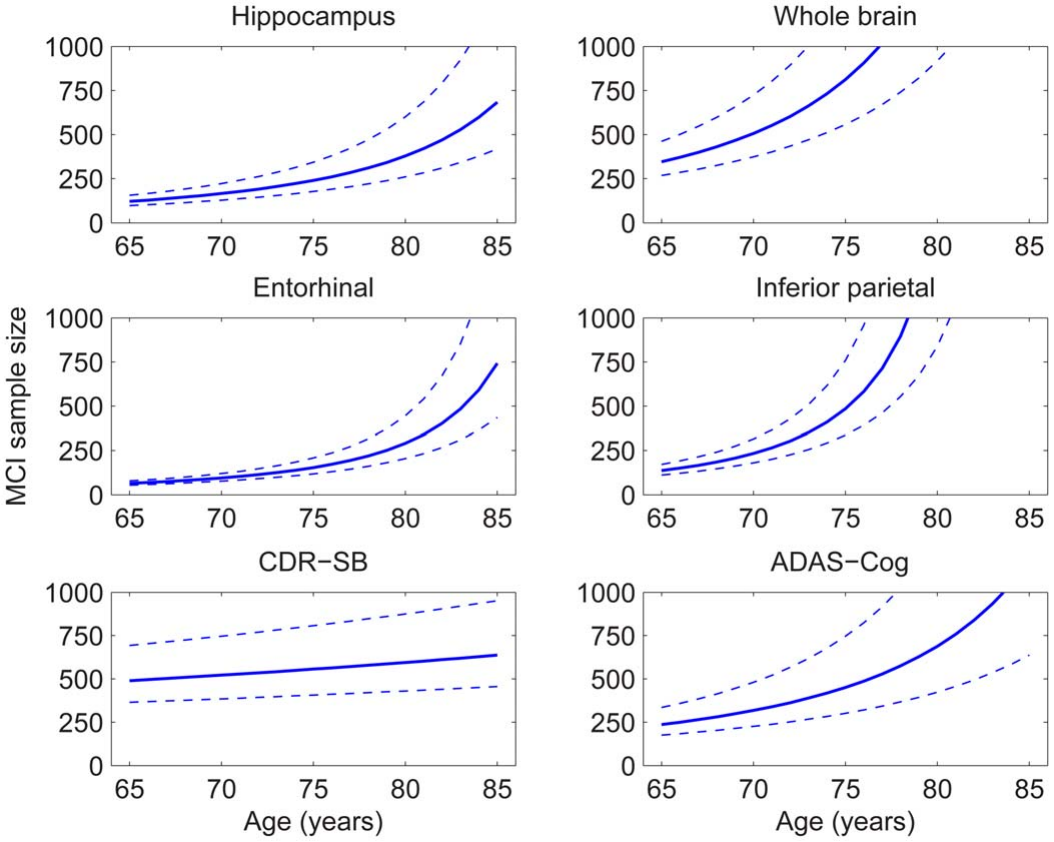
Estimated sample sizes with respect to age, per arm, to detect a 25% reduction in rate of change in MCI participants relative to age-matched change in HCs, at the p<0.05 level with 80% power assuming a 24 month trial with scans every six months. Sample sizes are estimated using a linear mixed effects model with fixed intercepts (no relative change at baseline) and random slopes and linear dependence on age applied to all data available up through 36 months. Dashed lines show the 95% confidence intervals.

**Figure 7:**
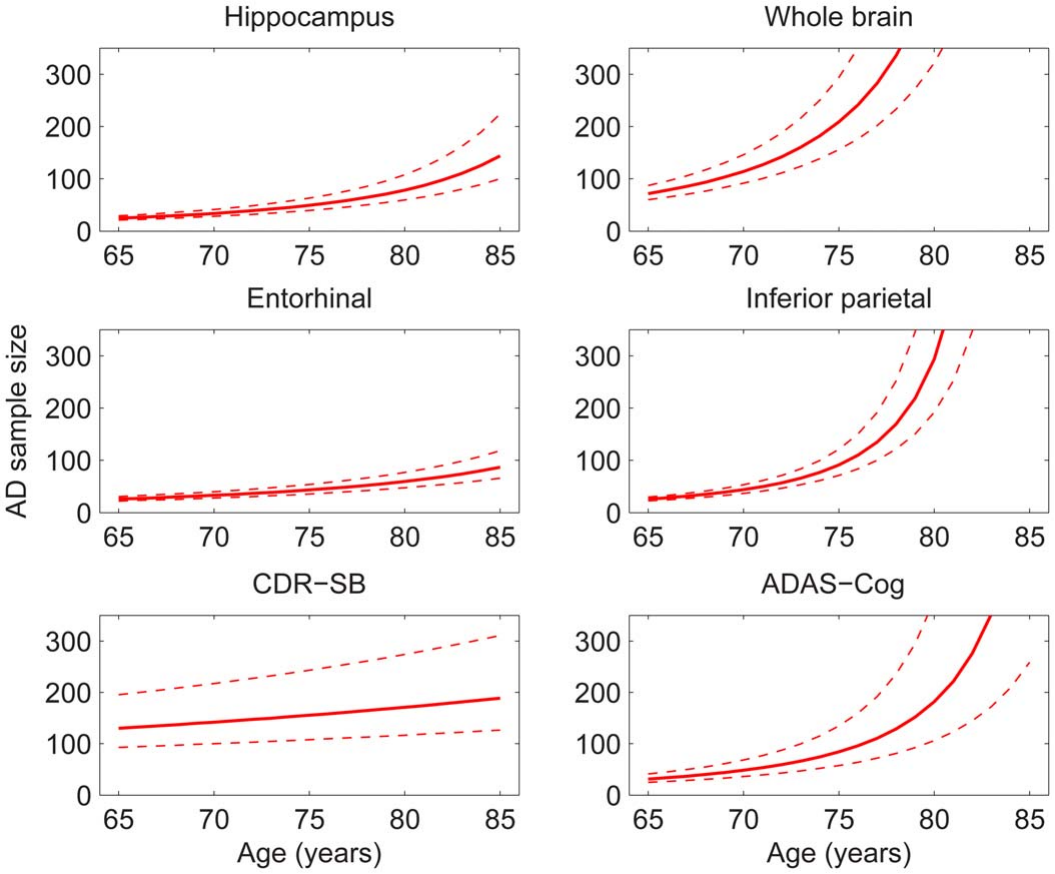
Estimated sample sizes, with respect to age, per arm, to detect a 25% reduction in rate of change in AD subjects relative to age-matched change in HCs. Other details are as described in the caption to Figure 6.

Figures 6 and 7 show sample size estimates (with approximate 95% CIs) as a function of baseline age for MCI and AD participants, respectively, using rates of change relative to age-matched HCs in neuroanatomical ROIs and standard clinical tests as the outcome measures [24]. For neuroanatomical measures, substantially smaller sample sizes are needed to effectively power a clinical trial at younger ages, while at older ages the sample size estimates rise sharply. However, the baseline age at which the sharp increase sets in depends on the ROI: e.g., ∼75 years for whole brain and inferior parietal, ∼80 years for hippocampus, and >85 years for entorhinal, in AD participants. This rapid increase in estimated sample size with age stems from the convergence pattern in atrophy rates seen in Figure 1. A similar pattern is observed with ADAS-Cog as the outcome measure, consistent with the convergence across groups in annual rate of decline shown in Figure 2. The age-dependence of sample size estimates is less marked for the CDR-SB, but estimated sample sizes are still larger for older as compared with younger cohorts. (The effect of reduction in unexplained random slope variance by including age as a random effect is described in File S1.).

### Potential Bias Due to Differential Dropout

It is possible that results could be affected by differential withdrawal rates. For example, the patterns of observed results could be explained if older patients with more severe symptoms withdrew after baseline from the study at a higher rate than younger subjects (e.g., due to increased morbidity or mortality). We therefore examined study discontinuation rates by age and symptom severity. Across MCI participants, 16 (4.6%) participants withdrew after the baseline session; these dropouts were distributed across the age ranges (2 aged 65–70; 7 aged 70–75; 2 aged 75–80; and 5 aged 80–85), with the exception that all MCI participants in the oldest age group completed at least one additional follow-up. For AD patients, 12 (7.7%) withdrew after the baseline session: 1 each in the 65–70 and 70–75 age groups, and 5 each in the 75–80 and 80–85 age groups.

There was no age-bias among the MCI participants who retained the diagnosis of MCI but who withdrew before the final (36-months) visit. For example, the ratio of the number of stable MCI participants with at least one followup but without a final visit, to the total number of MCI participants with at least one followup, for successive 5-year age groups between 65 and 90 years, was, respectively, 0.42, 0.44, 0.46, 0.46, and 0.57. Thus, differential dropout was unlikely to affect the estimated rate of progression to AD as a function of age.

Individuals who withdrew from the study after the baseline session did not differ significantly in symptom severity from those who completed one or more follow-up sessions. For example, MCI patients who withdrew after baseline showed a mean ADAS-Cog score of 11.1 (standard deviation, SD = 4.4) versus 11.6 (SD = 4.4; p = 0.65) for those who completed one or more follow-ups. For AD subjects, mean ADAS-Cog scores were 18.1 (SD = 4.3) and 19.9 (SD = 5.7; p = 0.24) for those who withdrew after baseline and those who completed at least one follow-up, respectively.

## Discussion

In this study, we present three lines of evidence indicating that AD proceeds more aggressively among younger elderly than among older elderly individuals, leading to a blurring of the distinction between AD and HC among the oldest old. First, we found that annual brain atrophy rates for AD and MCI individuals decrease with increasing baseline age, while atrophy rates for clinically normal older individuals remain constant or exhibit a slight increase with age. Second, we found that baseline CSF biomarker levels indicated greater disease burden in younger than in older MCI and AD patients, while disease burden increased with age among HCs. Third, we found that AD patients showed reduced rates of cognitive decline with increasing baseline age, despite uniformity of cognitive impairment at study entry. These findings have important implications for detection of AD among the oldest old, for the design of clinical trials of potentially disease-modifying therapies, and for biomarker and clinical disease trajectories.

All ROIs examined showed a decrease in longitudinal atrophy rate for MCI and AD individuals with increasing age at study entry. This decrease was most apparent in association cortices that tend to be affected later in the disease process, such as the inferior parietal lobule, middle temporal gyrus, and the retrosplenial cortex. The decrease in rate of atrophy with age in patient groups, combined with a trend toward increasing atrophy rates with age for HCs, resulted in a pattern of convergence of atrophy rates in association cortices across all diagnostic groups, with close convergence around 85 years of age, suggesting that in these brain regions neuronal number in patients is increasingly preserved with advancing baseline age [12]. However, the ROIs impacted by NFTs in earliest stages of the disorder (Braak stages I–II, transentorhinal, and stage III, limbic [38], [39]) continued to show significant differences in rates of decline for patients and older controls at more advanced ages, although differences were smaller than those observed at younger ages. Thus the power to discriminate MCI or AD individuals from HCs is differentially retained across ROIs, with the medial temporal ROIs that are particularly vulnerable to early neurofibrillary pathology retaining the strongest discriminative ability with advancing age.

In cross-sectional analyses, levels of disease burden as measured by CSF biomarkers decreased with increased age at study entry for MCI and AD participants, but increased with age for HC participants. The difference in the age-dependence of these biomarkers between patients and controls was particularly strong for ptau, leading to full convergence of ptau levels across patient groups at 85 years. The increase in ptau levels with age in HCs likely reflects increased burden of AD-specific neurofibrillary pathology [40], and may underlie the increase with age in rate of clinical decline [41] and atrophy rate for the entorhinal cortex and hippocampus [42] observed here for HCs.

Consistent with the structural MRI results, rates of longitudinal cognitive decline also showed convergence between patient and HC groups with increasing age at study entry. AD patients showed significant decrease in rate of decline on MMSE and a trend towards reduced rates of decline on ADAS-Cog with increased age, decreasing the difference in rates of decline between AD and HCs with increasing age. Due to uniform enrollment criteria across age, however, baseline cognitive measures did not differ with age within diagnostic groups, and showed constant separation among groups across age. This indicates that within each diagnostic group, individuals were at a uniform cognitive stage irrespective of baseline age. The differences observed in baseline CSF biomarkers and in rates of cognitive and structural change with age therefore do not stem from differences in clinical severity with age at the time of enrollment, but instead appear to reflect a decrease in rate of disease progression with baseline age. Within-cohort rates of decline for CDR-SB, however, were independent of age. CDR-SB measures global clinical or functional change, and may therefore not be as sensitive as ADAS-Cog to small declines in cognition [43]. A similar result was found in a recent study of age and rate of cognitive decline in AD [44], which also observed that older age at baseline was significantly associated with a slower rate of decline in ADAS-Cog 11, ADAS-memory, and MMSE. This decrease in rate of cognitive decline is consistent with our results showing decrease in 3-year rates of progression from MCI to dementia with advancing age: close to 50% of MCI participants aged 65 years developed dementia, whereas only about 25% of those aged 85 years did.

These findings showing a decrease with advancing age in multiple measures of neurodegeneration between AD patient cohorts and HCs are in agreement with earlier research showing that the correlation between dementia severity and NFT burden decreases with advancing age [45], and a more recent study assessing prevalence of moderate or severe pathological lesions in participants aged 69 to 103 years, which found attenuation with advancing age in the association between dementia and the densities of both neuritic plaques and NFTs in all examined ROIs [17]. In contrast, however, the latter study also found that dichotomized measures of neocortical and hippocampal atrophy allowed for distinguishing individuals with dementia from those without dementia, regardless of age, in agreement with the retained discriminative ability of hippocampus and entorhinal cortex measures shown here. Our findings are also in agreement with a recent cross-sectional analysis showing that the AD-related cognitive and structural MRI changes seen in AD patients aged 60–75 years are less salient in patients aged 80–91 years [18], and a recent longitudinal MRI analysis showing that rates of whole brain atrophy were greater in younger than older participants with amnestic MCI [46].

Our results showing smaller baseline structure size in older as compared with younger participants, modeled using linear fits with age, are in broad agreement with earlier manual and automated volumetry analyses [47]–[49]. In the latter study [49], covering the seventh through tenth decades in age, a generalized additive model (GAM) [50] was used, and nonlinear trends in baseline structure size as a function of age were reported. In the later decades, in particular, the cross-sectionally assessed atrophy accelerated or decelerated with age, depending on ROI and diagnostic group. It should be noted that cross-sectional analyses are not the most accurate approach to estimating longitudinal rates of change. It is also important to note that with GAMs the uncertainty in estimated structure size as a function of age can grow substantially from the middle of the age range toward the younger and older extremes in the age range modeled–which is often where the estimated nonlinearities tend to be most pronounced. This increased uncertainty may help account for discrepancies with other findings. For example, cross-sectional analysis comparing manual with voxel-based morphometry measures of hippocampal volume in healthy individuals [47] indicates acceleration of hippocampal atrophy with age in HCs. This is in agreement with our longitudinal results and in broad agreement with earlier results from manual longitudinal volumetry [51], [52], but is at variance with the deceleration of hippocampal atrophy with age in HCs resulting from the GAM analysis of cross-sectional data [49].

Our findings of reduced rates of clinical and morphometric decline with age in patients with AD may help shed light on the contradictory results that have been reported for incidence of AD dementia for the oldest old, aged 90 years and older. For example, the Cache County Study [53]–[55] and an autopsy study [56] found that incidence rates of AD decrease, beginning in the early 90 s. In contrast, the 90+ Study examining all-cause dementia, including 60% of participants diagnosed with AD, had a larger contingent of oldest participants, and found that dementia incidence continued to increase with age [57]. Although our results do not directly address the incidence of AD with age, our finding that rate of cognitive decline decreases with baseline age suggests that for the oldest old, clinical detection of AD, which relies on progressive decline in cognitive ability, may be more difficult. Reduction in CSF biomarkers of disease burden, and in atrophic changes in brain structure, with age suggests that the newly revised criteria for diagnosis of AD in research settings, which recommends incorporation of these biomarkers [58], may not help overcome this problem. It should also be noted, more broadly, that since incidence is the number of new cases in a population in a given time period, e.g. a year, non-constant rates of decline with age are likely to play a role in the incidence at different ages of clinically diagnosed AD.

The blurring of the distinction between HCs and patients with MCI or AD with advanced age has important implications for clinical trial design. As shown in Figures 6 and 7, all potential outcome measures evaluated here provide significant power for detecting disease-modifying therapeutic effects for MCI and mild AD participants aged 65–75, with the previously reported advantage for structural measures, such as entorhinal cortex and hippocampus, over clinical measures [24]. However, power is dramatically reduced with increasing age of the study sample. Due to the exponential rise in prevalence of AD with age (through approximately 85 years [16]), older individuals will increasingly be more available for clinical trials as compared with younger individuals. Yet, detecting a decrease in atrophy rate, or in rate of clinical decline, due to a disease-modifying therapy becomes increasingly difficult the older the study cohort. Thus, the extent to which older individuals are represented in the study sample could profoundly affect the power for detecting a therapeutic effect. In particular, a small but significant disease-modifying effect, which could significantly reduce the global burden of the disease [5], might be found in younger cohorts but would likely not be found in older cohorts. Given demographic trends, there is an urgent need to develop disease-modifying therapies to avert what will otherwise be an AD epidemic [59]–[61]. Since the power to detect therapeutic effects can be reduced dramatically in older cohorts, it is of immediate importance to consider fully cohort age in drug discovery.

With regard to disease trajectories, the observed reduction in rates of decline with advancing age allows for two distinct, plausible scenarios. In the first scenario, a cognitively healthy individual begins to experience a slightly elevated rate of decline at an early point in life, so that over a prolonged period, tissue loss gradually accumulates and cognitive function gradually declines. This slow course continues into advanced ages. At some point, despite the gradual nature of change, enough tissue loss and cognitive impairment has ensued to enable a physician to diagnosis the disorder.

In the second scenario, a cognitively healthy older individual initially experiences the same rates of structural and cognitive change as those of other cognitively healthy individuals of the same age, but the individual begins to experience a more rapid course of decline as symptoms develop and worsen. Rate of decline then slows at some point, so that at an advanced age, the rate of decline again approaches the rate observed for healthy older individuals of the same age. In this scenario, the biomarker trajectory follows a sigmoidal course. Despite the popularity of sigmoidal curves for describing biomarker trajectories in AD [62]–[65], and the development of mathematical models of neuron death kinetics where neuron death is considered not to arise from cumulative damage [66], [67], there is no conclusive evidence demonstrating a slowing in rate of brain atrophy over time within individual AD patients. Furthermore, slowing in rate of neuronal loss from age and disease progression might not be biologically plausible: arguments from protein homeostasis indicate instead that cell death is more likely to accelerate than to slow with disease progression [15].

A recent investigation [68] of the shapes of AD biomarker trajectories examined whether, as suggested by sigmoidal models, hippocampal atrophy rate slowed with increasing disease severity in four patient cohorts. Employing a liberal significance threshold (p-value  = 0.1), and restricting the sample to amyloid-positive participants, evidence of slowing of the rate of volume loss with increasing disease severity, as assessed with MMSE score, was found in three cohorts when cross-sectional data were analyzed but not when longitudinal data were analyzed. In the fourth cohort the reverse occurred: evidence of slowing of the rate of volume loss with increasing disease severity was found when longitudinal data were analyzed but not when cross-sectional data were analyzed. These inconsistencies do not strongly support the sigmoidal model for AD-related atrophy. Furthermore, when the sample was not restricted to amyloid-positive participants, cross-sectional analyses consistently showed an increase in hippocampal atrophy with increasing disease severity, and longitudinal analyses consistently showed an increase in hippocampal atrophy rate with increasing disease severity–results that do not support the contention that atrophy rates eventually decelerate with advancing disease severity.

Another possible explanation of differences in rates of decline with age observed here is differential dropout, in which older patients with more severe decline dropped out of the study at a higher rate than younger individuals with more severe decline. However, evidence suggests that such differential dropout is unlikely to have affected the observed results. Older participants were not more likely than younger participants to drop out after the baseline session, and there was no difference in symptom severity between those who dropped out after baseline versus those who completed one or more follow-ups. However, differential recruitment into the study may contribute to the observed results. Participation in a trial such as ADNI requires a large commitment of time and effort. It is possible that for the oldest old patients with AD, if they were experiencing a rapid course of decline, they or their caregivers may have been less willing to participate in a burdensome, non-treatment study than those with milder decline. Such differential enrollment, where younger but not older rapidly declining individuals enrolled in the study seems unlikely given the systematic decrease in rates of decline with increasing age for both MCI and AD cohorts.

Limitations of this study include the highly select nature of the study sample: individuals were required to be generally healthy with no evidence of comorbid disorders that could affect study participation, such as depression or vascular dementia. A second limitation is the lack of histopathological verification of AD pathology. It is possible that many participants suffer from subclinical cerebrovascular disease, and the contribution of cerebrovascular disease to dementia symptoms may be greater among the very old. A recent neuropathological study [69], in addition to finding attenuation in the association between dementia and NFT burden in nonagenarians, also found an increase in vascular burden in mixed pathologies in people over 90 years. This increases the possibility of misdiagnosis, particularly since microvascular pathology and AD are synergistic in the development of dementia in old age [70], [71]. Furthermore, and somewhat circularly, the convergent patterns in rates of decline might themselves contribute to increased potential for misdiagnosis at older ages. The small number of AD individuals over the age of 85 years is a further limitation of the study.

Despite these limitations, this study suggests that the association between neuropathological markers and dementia attenuates with age and needs to be taken into account in models of AD. The degree of attenuation is remarkably uniform across diverse markers–CSF AD-related protein densities, atrophy rates in multiple brain regions, and rates of clinical decline–all showing strong convergence patterns across diagnostic groups at older ages, though it is significant that atrophy rates in the hippocampus and particularly the entorhinal cortex indicate slower convergence. As a result, methods for early disease detection and assessment of therapeutic interventions cannot be applied uniformly across the entire elderly age spectrum. Given demographic trends, in particular the rapid growth in the proportion of very old individuals, greater emphasis needs to be placed on further elucidating the effects of age on the disease process to better prepare for the diagnosis, care, and treatment of the oldest old.

## Supporting Information

File S1 (DOC)
