# Supplementary material for: Rates of Decline in Alzheimer Disease Decrease with Age
Source: PLoS One. 2012 Aug 2;7(8):e42325. doi: 10.1371/journal.pone.0042325 (PMC3410919; doi:10.1371/journal.pone.0042325)
Supplement: File S1 — (DOC) [file pone.0042325.s001.doc]

**Supporting Information**

REDUCTION IN UNEXPLAINED RANDOM SLOPE VARIANCE

Inclusion of age as a random effect reduced the “unexplained” variance σ_m_^2^ in the random slopes. Due to the stronger age-effect, reductions were greater for AD than for MCI. For example, the percentage of random slope variance explained by age was: hippocampus 24% AD (0% MCI); entorhinal 18% (3.5%); middle temporal 19% (5%); whole brain 16% (3%); inferior parietal 26% (6%); retrosplenial cortex 25% (6%). Comparing the sample size estimates for the AD cohort as a whole when not modeling for age, and those that result for the mean cohort age when modeling for age, illustrates the benefits of reduced variance when including age as a covariate. For example, the mean baseline age for the AD cohort was 75.8 years. Sample size estimates [95% confidence interval] for the full cohort (ignoring age as a covariate), and for the mean baseline age (including age as a covariate) were respectively: hippocampus 90 [71 117], 59 [67 110]; entorhinal 54 [44 67], 40 [32 50]; whole brain 200 [142 303], 148 [109 213].

ADNI

Data used in the preparation of this article were obtained from the Alzheimer’s Disease Neuroimaging Initiative (ADNI) database (adni.loni.ucla.edu). The ADNI was launched in 2003 by the National Institute on Aging (NIA), the National Institute of Biomedical Imaging and Bioengineering (NIBIB), the Food and Drug Administration (FDA), private pharmaceutical companies and non-profit organizations, as a $60 million, 5-year public- private partnership. The primary goal of ADNI has been to test whether serial magnetic resonance imaging (MRI), positron emission tomography (PET), other biological markers, and clinical and neuropsychological assessment can be combined to measure the progression of mild cognitive impairment (MCI) and early Alzheimer’s disease (AD). Determination of sensitive and specific markers of very early AD progression is intended to aid researchers and clinicians to develop new treatments and monitor their effectiveness, as well as lessen the time and cost of clinical trials.

The Principal Investigator of this initiative is Michael W. Weiner, MD, VA Medical Center and University of California – San Francisco. ADNI is the result of efforts of many co- investigators from a broad range of academic institutions and private corporations, and subjects have been recruited from over 50 sites across the U.S. and Canada. The initial goal of ADNI was to recruit 800 adults, ages 55 to 90, to participate in the research, approximately 200 cognitively normal older individuals to be followed for 3 years, 400 people with MCI to be followed for 3 years and 200 people with early AD to be followed for 2 years. For up-to-date information, see www.adni-info.org.

PARTICIPANTS

The ADNI general eligibility criteria have been described elsewhere (Petersen RC, Aisen PS, Beckett LA, Donohue MC, Gamst AC, Harvey DJ, et al. Alzheimer's Disease Neuroimaging Initiative (ADNI): clinical characterization. Neurology. 2010; **74**(3): 201-9). Briefly, participants are not depressed, have a modified Hachinski score of 4 or less, and have a study partner able to provide an independent evaluation of functioning. HC participants have a Clinical Dementia Rating (CDR) of 0. Participants with MCI have a subjective memory complaint, objective memory loss measured by education-adjusted scores on Wechsler Memory Scale Logical Memory II, a CDR of 0.5, preserved activities of daily living, and absence of dementia. Participants with AD have a CDR of 0.5 or 1.0 and meet National Institute of Neurological Disorders and Stroke and Alzheimer's Disease and Related Disorders Association criteria for probable AD.
